# Inhibition of the Hantavirus Fusion Process by Predicted Domain III and Stem Peptides from Glycoprotein Gc

**DOI:** 10.1371/journal.pntd.0004799

**Published:** 2016-07-14

**Authors:** Gonzalo P. Barriga, Fernando Villalón-Letelier, Chantal L. Márquez, Eduardo A. Bignon, Rodrigo Acuña, Breyan H. Ross, Octavio Monasterio, Gonzalo A. Mardones, Simon E. Vidal, Nicole D. Tischler

**Affiliations:** 1 Molecular Virology Laboratory, Fundación Ciencia & Vida, Santiago, Chile; 2 Laboratory of Structural Cell Biology, Department of Physiology, and Center for Interdisciplinary Studies of the Nervous System (CISNe), Universidad Austral de Chile, Valdivia, Chile; 3 Laboratorio de Biología Estructural y Molecular, Facultad de Ciencias, Universidad de Chile, Santiago, Chile; University of Texas Medical Branch, UNITED STATES

## Abstract

Hantaviruses can cause hantavirus pulmonary syndrome or hemorrhagic fever with renal syndrome in humans. To enter cells, hantaviruses fuse their envelope membrane with host cell membranes. Previously, we have shown that the Gc envelope glycoprotein is the viral fusion protein sharing characteristics with class II fusion proteins. The ectodomain of class II fusion proteins is composed of three domains connected by a stem region to a transmembrane anchor in the viral envelope. These fusion proteins can be inhibited through exogenous fusion protein fragments spanning domain III (DIII) and the stem region. Such fragments are thought to interact with the core of the fusion protein trimer during the transition from its pre-fusion to its post-fusion conformation. Based on our previous homology model structure for Gc from Andes hantavirus (ANDV), here we predicted and generated recombinant DIII and stem peptides to test whether these fragments inhibit hantavirus membrane fusion and cell entry. Recombinant ANDV DIII was soluble, presented disulfide bridges and beta-sheet secondary structure, supporting the *in silico* model. Using DIII and the C-terminal part of the stem region, the infection of cells by ANDV was blocked up to 60% when fusion of ANDV occurred within the endosomal route, and up to 95% when fusion occurred with the plasma membrane. Furthermore, the fragments impaired ANDV glycoprotein-mediated cell-cell fusion, and cross-inhibited the fusion mediated by the glycoproteins from Puumala virus (PUUV). The Gc fragments interfered in ANDV cell entry by preventing membrane hemifusion and pore formation, retaining Gc in a non-resistant homotrimer stage, as described for DIII and stem peptide inhibitors of class II fusion proteins. Collectively, our results demonstrate that hantavirus Gc shares not only structural, but also mechanistic similarity with class II viral fusion proteins, and will hopefully help in developing novel therapeutic strategies against hantaviruses.

## Author Summary

The infection of cells by enveloped viruses involves the fusion of membranes between viruses and cells. This process is mediated by viral fusion proteins that have been grouped into at least three structural classes. Membrane-enveloped hantaviruses are worldwide spread pathogens that can cause human disease with mortality rates reaching up to 50%, however, neither a therapeutic drug nor preventive measures are currently available. Here we show that the entrance of Andes hantavirus into target cells can be blocked by fragments derived from the Gc fusion protein that are analogous to inhibitory fragments of class II fusion proteins. The Gc fragments acted directly over the viral fusion process, preventing its late stages. Together, our data demonstrate that the hantavirus Gc protein shares not only structural, but also mechanistic similarity with class II fusion proteins, suggesting its evolution from a common or related ancestral fusion protein. Furthermore, the results outline novel approaches for therapeutic intervention.

## Introduction

The genus *Hantavirus* of the family *Bunyaviridae* comprises diverse viruses that are highly pathogenic to humans. In Asia and Europe the Hantaan, Seoul and PUUV viruses cause hemorrhagic fever with renal syndrome, while in America ANDV and Sin Nombre virus can lead to hantavirus pulmonary syndrome with mortality rates above 30% [1–5]. Hantaviruses are currently the most lethal human pathogenic viruses known to occur in America and, due to the lack of Food and Drug Administration (FDA)-approved preventive or therapeutic measures [6, 7], they have been classified as category A pathogens. Like other members of the *Bunyaviridae* family, hantaviruses have a tri-segmented single strand RNA genome of negative polarity packaged by the nucleoprotein into viral ribonucleocapsids, which are also associated to the viral RNA-dependent RNA polymerase [8]. A lipid membrane, which further envelopes the viral ribonucleocapsids, anchors the viral Gn and Gc glycoproteins. This viral envelope is derived from a host cell membrane during the budding process of the virus [9, 10]. To infect new cells, hantaviruses bind to cell surface receptors [11–14], and are subsequently taken up by endocytosis [15, 16]. A crucial step in viral cell entry is the fusion of the virus with an endosomal membrane of the host, escaping from its degradation in lysosomes. Yet, little is known about the fusion process of hantaviruses; however, our recent data show that the low pH of endosomes triggers a non-reversible fusion process, in which the Gc protein inserts into target membranes and forms a highly stable post-fusion homotrimer [17].

In general, virus-cell membrane fusion is thought to be accomplished by multiple steps [reviewed in 18, 19]. After the activation, viral fusion proteins insert a fusion peptide or fusion loop into a target membrane. At this intermediate stage, the fusion peptide is located at one end of the fusion protein while the opposite end is anchored to the viral envelope membrane by a transmembrane region, thereby bridging the viral and cellular membranes. By undergoing additional conformational changes, the fusion protein is thought to pull both anchors together, until it reaches a hairpin-like conformation in which both membrane-inserted domains are located at the same end of the protein. Once the opposed membranes have been brought into a close distance by the introduction of local membrane curvature, the fusion of the outer leaflets of the membranes produces a hemifusion intermediate, followed by the full fusion of the membranes. The fusion culminates in the formation of a fusion pore through which the virus can deliver its ribonucleocapsids into the cell cytosol to initiate replication.

Viral fusion proteins are currently grouped into at least three different classes based on their molecular structures: class I fusion proteins have a high alpha helical content, class II proteins consist principally of beta sheets, while class III fusion proteins include characteristics from the first two classes [reviewed in 19–21]. Early *in silico* and *in vitro* analyses suggested that the Gc glycoprotein of hantaviruses shares structural similarity with class II fusion proteins [22, 23]. This notion has also been proposed for other members of the *Bunyaviridae* [24–28], and the crystal structure of Gc from Rift Valley Fever virus (RVFV) ultimately confirmed this notion for phleboviruses [29].

Class II fusion proteins are composed of three domains (I-III) and a stem region that connects the ectodomain to the transmembrane anchor [30–34]. To adopt a hairpin-like structure, DIII moves towards the fusion loop [35, 36], while the stem region, which is connected to the transmembrane anchor, is thought to follow the movement of DIII by folding against the trimeric core formed by the fusion protein [37–39]. The extensive conformational changes that occur during the fusion process offer opportunities to disrupt the fusion cascade, thereby blocking viral infection. Ligands that bind selectively to an intermediate form of the fusion protein preceding its post-fusion conformation can delay or inhibit viral entry. In the case of human immunodeficiency virus 1, which has a fusion protein with a class I fold, there is a licensed drug based on a 20-residue peptide [reviewed in 40, 41]. This peptide comprises a partial sequence of the outer layer of the trimeric post-fusion hairpin conformation of gp41 and binds to the trimeric core of the fusion protein in its extended intermediate conformation, preventing the foldback reaction [reviewed in 42, 43]. Among class II proteins, DIII and the stem region form the outer layer of the trimeric post-fusion conformation [35–37]. Liao & Kielian (2005) showed that the addition of soluble DIII with or without the stem region of Semliki Forest virus E1 protein and soluble DIII of Dengue virus type 2 (DV2) E protein inhibit the entry of the respective virus into cells and confirmed a common inhibitory mechanism of class I and class II fusion proteins [44]. Other studies have shown that peptides derived from the stem region of the fusion protein of flavi- and phleboviruses also inhibit viral cell entry [45, 46]. The binding of stem peptides to fusion proteins is thought to prevent the post-fusion conformation as in the case of DIII; however, their amphipathic characteristics seem to allow binding to the virus before attachment to the cell, and are hence thought to be carried on the virus into endosomes [47, 48]. This characteristic provides an advantage for the delivery of the inhibitor to the site of virus-cell membrane fusion when this process occurs in a closed endosomal compartment. Here, we hypothesized that if hantavirus Gc shares mechanistic similarity with class II fusion proteins, then it should be inhibited with strategies used for other class II fusion proteins. To test this hypothesis, we predicted and produced DIII and the stem region of ANDV Gc and assessed them for ANDV inhibition. Our results show that indeed both, recombinant DIII and synthetic stem peptides, interfered with the ANDV infection, acting at late stages of the ANDV fusion process.

## Materials and Methods

### Preparation of DIII expression plasmids

For the PCR amplification of the predicted DIII and DIIIS sequences we used the cloned cDNA of the M segment from ANDV isolate CHI-7913 [49] and from PUUV strain K27 cloned into pWRG/PUUV-M(s2) expression plasmid [50] (kindly provided by Jay Hooper, USAMRIID, USA), GenBank accession numbers AAO86638 and L08754, respectively. The PCR products were cloned into pET28a, which gave rise to fusion proteins with an N-terminal tag of 34 residues including polyhistidine (His-tag). For the preparation of DIII without the His-tag, the PCR product of ANDV DIII was cloned into pGST-Parallel-1 [51]. The expression product of this plasmid contained an N-terminal tag of 314 residues including a Glutathione S transferase (GST) affinity tag followed by a cleavage site for the tobacco etch virus (TEV) protease. After cleavage with TEV protease, 7 residues from the GST-fusion protein remained fused to the N-terminal of DIII, corresponding to the sequence GAMDPEF.

### Expression and purification of DIII proteins

The expression of recombinant DIII proteins was performed as established before [52]. In brief, His-tagged fusion proteins were expressed in *Escherichia coli* (*E*.*coli*) BL21, which was transformed with the different pET28a/DIII plasmids. The isopropyl-beta-D-thiogalactopyranoside-induced bacteria were lysed by sonication in buffer containing 20 mM Tris, 50 mM NaCl, and 0.2 mM phenylmethanesulfonylfluoride, pH 8. Lysis was performed by sonication on ice and soluble proteins were purified through the ion exchange resin Bio-Rex 70 (Bio-Rad Laboratories) followed by tandem ultrafiltration on devices with 30 kDa and 3 kDa of molecular weight cut-off. Recombinant, untagged DIII was produced following a similar purification procedure described elsewhere [53], with some modifications. Briefly, *E*. *coli* Origami 2(DE3) (Novagen), were transformed with pGST-Parallel-1/DIII, and expression was induced, and bacteria were pelleted and lysed as described above. The clarified supernatant was purified on glutathione-Sepharose 4B (GE Healthcare), and the bound protein was eluted with 20 mM reduced glutathione (Sigma-Aldrich). His6-tagged TEV protease was used to cleave off the GST moiety, and the GST moiety and the TEV protease were removed by sequential passage through glutathione-sepharose 4B and Ni-NTA (Qiagen) resins. Eventually, DIII was concentrated using an ultrafiltration device with 3 kDa cut-off, and further purified on a HiLoad 16/60 Superdex 200 prep grade column (GE Healthcare).

### DIII protein analysis

Proteins were analyzed by standard Tris-glycine SDS-polyacrylamide gel electrophoresis using 4–12% or 15% gels. For irreversible reduction, 8 mM dithiothreitol was added, the proteins were heated to 95°C for 3 min, and subsequently alkylated by incubation with 24 mM iodoacetamide for 15 min at 37°C. Circular dichroism measurements were performed using a spectropolarimeter (Jasco-J600), and 5 scans were recorded at room temperature between 190 and 260 nm, using a 1 mm optical pass cuvette. Measured values of ellipticity were converted into ellipticity per amino acid residue. For deconvolution of the spectra, different databases of CONTIN and CDSSTR servers [54–56] were used.

### Peptides

Peptides spanning the predicted stem region of ANDV Gc were synthesized (New England Peptides). The N-terminal R1 peptide comprised the sequence HLERVTGFNQIDSDKVYDDGAPP, and the C-terminal R2 peptide comprised the sequence TFKCWFTKSGEWLLGILNGN. Two additional peptides were used, R2.1 and R2.2, comprising either the first ten residues or the last ten residues of the R2 peptide, respectively. To avoid the introduction of additional charges, the C-terminal of R2.1 was amide-modified, and the N-terminal of R.2.2 was acid-modified, comprising the sequences TFKCWFTKSG and EWLLGILNGN, respectively. The NN peptide comprising the sequence QLVTARQKLKDAEKAVEVDPDDVNKSTLQRRAAVSTLETKLG, derived from the ANDV nucleoprotein, was used as control. Such nucleoprotein-based peptide was chosen to limit the possibility of inter- or intramolecular interactions with the fusion protein that may occur during its conformational changes in the fusion process. This NN peptide of 42 residues was used as control of both short R peptides and longer DIII fragments. All peptides were soluble in HNE buffer (10 mM HEPES, 130 mM NaCl, 0,1 mM EDTA, pH 7.4), except peptides R2 and R2.2, which were dissolved in 10 mM borate buffer (pH 9).

### Virus and cells

ANDV isolate CHI-7913 (kindly provided by Héctor Galeno, Instituto de Salud Pública, Chile) was propagated in Vero E6 cells (ATTC) as described before [57]. All work involving the infectious virus was performed under biosafety level 3 conditions (Centro de Investigaciones Médicas, Pontificia Universidad Católica de Chile, Chile). 293FT cells (Invitrogen) were propagated in DMEM supplemented with 10% fetal calf serum (FCS). CHO-K1 cells (ATTC) were grown in F12-K medium containing 10% FCS.

### ANDV infectivity titration

The infection of Vero E6 cells by ANDV (multiplicity of infection (MOI) = 0.1) was quantified by flow cytometry as formerly established [57]. Briefly, cells were incubated with ANDV for 1 h at 37°C in the presence and absence of protein or peptide inhibitor candidates. Subsequently, cells were washed and infection was allowed to proceed for 16 h. Cells were next fixed with 2% (w/v) paraformaldehyde for virus inactivation, and permeabilized with 0.1% Triton X-100. For immunofluorescence staining, cells were incubated for 45 min with anti-ANDV N monoclonal antibody (mAb) 7B3/F6 [58] and then the primary antibody was detected with goat anti-mouse immunoglobulin conjugated to Alexa 555 (Life Technologies). ≥10.000 cells of each condition were analyzed using a flow cytometer (FACS CAN II, Becton Dickinson). The standard deviation of at least n = 3 experiments is indicated as the error bar of each value.

### Production of simian immunodeficiency virus (SIV) vectors pseudotyped with vesicular stomatitis virus (VSV) G protein or ANDV glycoproteins and transduction

SIV vectors bearing the VSV glycoprotein were prepared as indicated elsewhere [59]. Briefly, 293FT cells were transfected with 8 μg of plasmid coding for SIV Gag-Pol (pSIV3+), 8 μg of plasmid encoding GFP as an RNA minigenome (pGAE1.0) [60], and 4 μg of plasmid coding for the envelope protein G of VSV (pVSV-G, Clontech). Alternatively, the plasmid pI.18/GPC coding for ANDV glycoproteins was used to generate SIV vectors pseudotyped with ANDV glycoproteins. At 48 h post-transfection, supernatants containing pseudotyped particles were concentrated by centrifugation at 100,000 g for 75 min. Different dilutions of VSV-G pseudotyped SIV vectors were incubated for 1 h with Vero E6 cells in the presence and absence of protein or peptide inhibitor candidates. Three days later, the expression of GFP in transduced cells was analyzed by flow cytometry (FACScan, Becton Dickinson). ≥10.000 cells were counted for each experimental condition.

### Cytotoxicity

A cell proliferation assay was used to assess cytotoxicity of the Gc domains and peptides as described by the manufacturer (CellTiter96, Promega). Briefly, Gc domains and peptides were incubated with Vero E6 cells for 18 h at 37°C, and the conversion of tetrazolium to formazan was assessed by measuring the absorbance at 570 nm in a microplate reader (Synergy 4, BioTek).

### Cell-cell fusion assay

A three-color based cell-cell fusion assay was performed as established before [22]. Briefly, 48 h after the transfection of Vero E6 cells with pI.18/GPC [59] or pWRG/PUUV-M(s2) [50] coding for the glycoproteins of ANDV and PUUV, respectively, the cells were incubated with low pH media (E-MEM, 20 mM sodium succinate, pH 5.5) for 5 min at 37°C. Three hours later, the cell cytoplasm was stained with 5-chloromethylfluorescein diacetate (CellTracker Green CMFDA, Invitrogen), cell nuclei with DAPI (Life Technologies), and Gc was labeled by anti-Gc mAb 2H4/F6 [61], and anti-mouse immunoglobulin conjugated to Alexa555 (Invitrogen). The fusion index of Gc-expressing cells was calculated by the formula:

$$Fusion\;index\;=\;1\;-\;\left[\frac{number\;of\;cells}{number\;of\;nuclei}\right]$$ (1)

For each sample, approximately 200 nuclei per field were counted (20x magnification), and the mean fusion index of five fields was calculated (n = 3).

### ANDV infection mediated by virus-plasma membrane fusion

Vero E6 cells were pre-chilled on ice for 10 min with 20 mM NH_4_Cl. The adsorption of ANDV (MOI = 0.2) was performed at 4°C for 1h. Next, cells were washed, and the fusion of the virus with the plasma membrane was triggered by incubation in low pH media (E-MEM, 20 mM sodium succinate, pH 5.5) for 5 min at 37°C in the presence and absence of inhibitor candidates. Next, cells were washed, and infection was followed by incubation for 16 h at 37°C in the presence of 20 mM NH_4_Cl. Subsequently, viral infection was assessed as described above.

### Acid-induced Gc homotrimerization

The multimerization state of Gc was assessed by sucrose gradient centrifugation as previously established [17]. Briefly, ANDV was treated at pH 5.5 to allow for the rearrangement of glycoproteins on the viral envelope. Where indicated, DIII or R2 were added to the virus during its low pH incubation during 30 min at 37°C. Subsequently, viral glycoproteins were extracted by 1% Triton X-100 and separated in a gradient of 7–15% (w/v) sucrose by centrifugation at 150,000 g for 16 h. Fractions were collected, and the presence of Gc in each fraction was assessed by western blot analysis using anti-Gc mAb 2H4/F6 [61]. The molecular mass of each fraction was assessed by the Coomassie staining of a molecular marker (Gel filtration standard, Bio-Rad) that was applied to the same sedimentation gradient. The experimental molecular mass of the marker was next plotted against the log of its theoretical molecular mass in the panel above the western blots of the gradient. The stability of the Gc homotrimer was further tested by trypsin digestion as indicated before [17]. Briefly, well-characterized VLPs projecting ANDV glycoproteins were prepared as described elsewhere [62] and were incubated for 30 min at pH 5.5. After the acidification, VLPs were incubated with TCPK trypsin (Sigma) for the indicated times. Finally, digestion was stopped by adding sample buffer and heating to 95°C for 10 min. The digestion of Gc was tested by western blot analysis, using anti-Gc mAb as described above.

### Cell-based hemifusion assay

In this assay, the transfer of monosialotetrahexosylganglioside (GM1) from an effector cell to a target cell was measured as described before for SNARE proteins [63]. To adapt this assay to measure the ANDV glycoprotein-mediated GM1 transfer, 293FT cells (GM1^+^) were transfected with pI.18/GPC [59] using Lipofectamine 2000 (Invitrogen), as indicated by the manufacturer. Forty-five h post-transfection, the cells were detached from the plates, and resuspended in supplemented DMEM. At the same time, target CHO-K1 cells (GM1^-^) were trypsinized and labeled with 40 μM 7-amino-4-chloromethylcoumarin (CellTracker Blue CMAC, Invitrogen) in supplemented 12-K medium for 40 min at 37°C. The excess dye was then removed by replacing the dye-containing media with supplemented F12-K medium and subsequent incubation for 30 min. After washing with PBS, target cells were resuspended in supplemented DMEM. Next, effector cells (transfected 293FT cells) and CMAC-labeled target cells (CHO-K1 cells) were combined in a ratio of 1:4 (effector:target) for 3 h. Then, the fusion protein was activated by incubating the cells in low pH media (DMEM, sodium succinate 20 mM, 10% FCS, pH 5.5) for 5 min at 37°C. This medium was replaced with supplemented DMEM, pH 7.4 and after 30 min of incubation the cells were fixed with 4% paraformaldehyde. For fluorescence staining, cells were incubated for 30 min with 5 μg/ml of cholera toxin β-subunit (CTX) conjugated to Alexa Fluor 488 (Invitrogen) at 37°C, washed with PBS, and mounted with DABCO. Cells were examined by confocal microscopy (Fluorview FV1000, Olympus and single plane images from 10 different microscopic fields were taken in each condition. The percentage of GM1 transfer was calculated using the formula:

$$\%\;GM1\;transfer\;=\frac{N_{a}}{N_{b}\cdot 100}$$ (2)

where N_a_ corresponds to the number of cells positive for CMAC and GM1, while N_b_ corresponds to the number of cells positive for GM1 that are furthermore in contact with at least one target cell. Cells were considered positive for GM1 transfer when the fluorescent label was detected at the full circumference of the cells. The standard deviation of at least n = 3 experiments was calculated and was indicated as an error bar for each value.

### Statistical analysis

A Student´s t-test was performed for statistical evaluation of n≥3 independent epxeriments: ***, P < 0.00025; **, P < 0.0025; *, P < 0.025.

## Results

### Prediction of DIII and stem fragments with inhibitory potential

**Fig 1:**
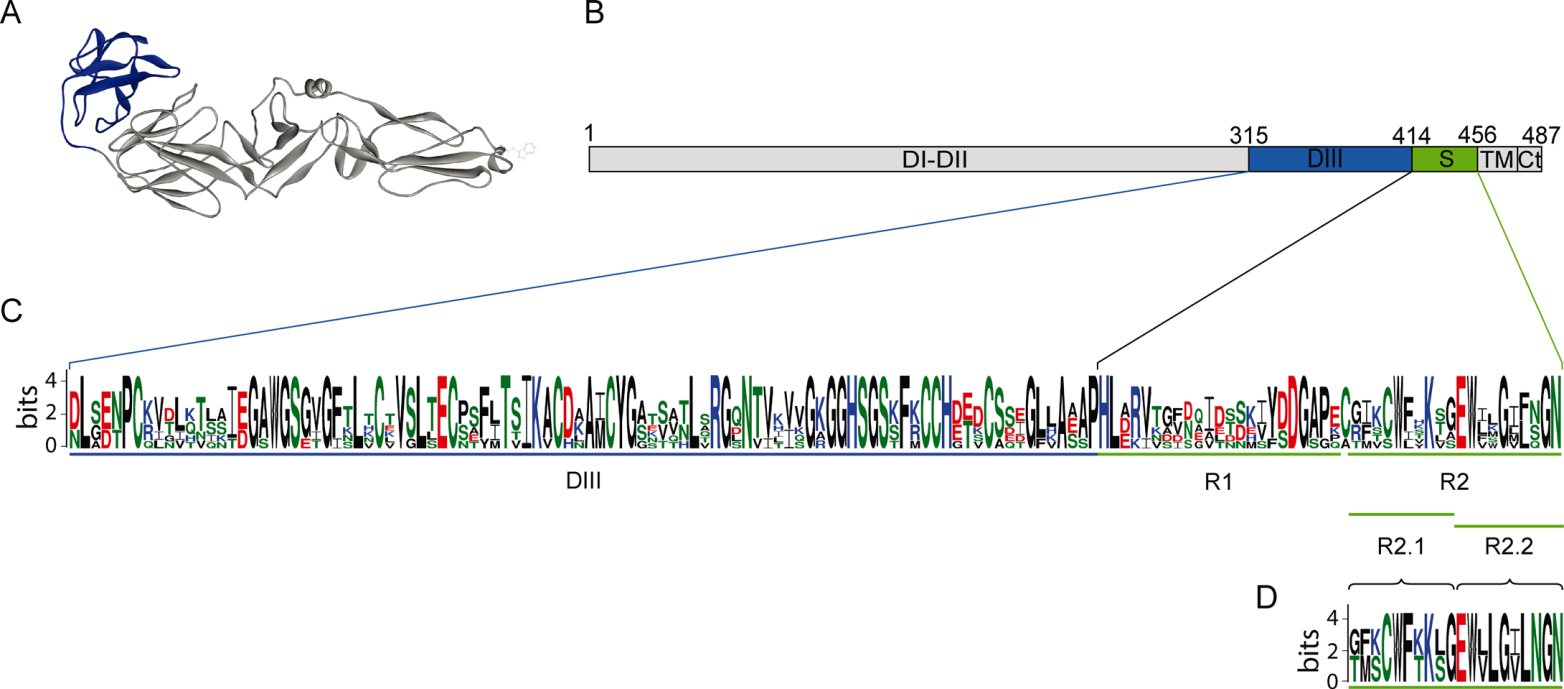
Localization of predicted DIII and stem fragments in ANDV Gc. (A) Molecular model for ANDV Gc in its pre-fusion conformation [23] based on the TBEV E protein (PDB: 1SVB) [31]. The predicted DIII is indicated in blue and the predicted domains I-II are shown in grey. (B) Scheme of the ANDV Gc primary structure indicating predicted residues for DIII (blue) and the predicted stem region (green). S indicates the stem region; TM indicates the transmembrane region; Ct indicates the cytoplasmic tail. (C) Sequence logo representation of a multiple sequence alignment including DIII and stem regions of the genus *Hantavirus* built with WebLogo [65]. The sequence conservation at each position is represented by the overall height of the stack at that position, while the conservation of a specific residue at that position is given by the height of the symbols. Acid residues are indicated in red, basic residues in blue, apolar residues in black and polar residues in green. For the multiple sequence alignment, glycoprotein sequences were retrieved from GenBank representing the four hantavirus phylogroups [66] from different reservoirs, including rodent-borne hantaviruses from the subfamiliies *Avicolinae* (PUUV; accession no. NP_941983.1), *Murinae* (HTNV; accession no. NP_941978.1), *Sigmodontinae* (ANDV; accession no. AAO86638.1), and also hantaviruses harbored in shrews, moles and bats from the families *Soricidae* (Thottapalayam virus; accession no. ABU82619.1), *Talpidae* (Asama virus; accession no. ACI28508.1), and *Rhinolophidae* (Longquan virus; accession no. AGI62348.1). The regions spanned by recombinant domains and peptides are indicated schematically below the logo representation by DIII, R1, R2, R2.1 and R2.2. (D) Sequence logo representation of a sequence alignment from the partial stem region of ANDV and PUUV Gc.

For the hantavirus Gc protein, we predicted DIII and the stem region by sequence alignments with known class II fusion proteins and subsequent model derivation. These proteins included among others the more recently crystallized RVFV Gc [29]. None of the new sequence alignments achieved a higher sequence identity, greater cysteine match or model validation scores compared to the alignment used for the original Gc model structure [23], which is based on the pre-fusion structure of the tick-borne encephalitis virus (TBEV) E protein (PDBid: 1SVB) [31]. For this reason, we used the alignment from the original ANDV Gc model [23] to identify a putative DIII in ANDV Gc (Fig 1A and 1B). The sequence that was derived from this model (residues Asp315-Leu414) was termed ANDV DIII, and served as template to predict a putative DIII in Gc of other hantaviruses such as PUUV. We subsequently defined the putative stem region in ANDV Gc as the sequence encompassing residues Leu414-Asn456, which corresponded to the region between the C-terminal end of the predicted DIII and the predicted Gc transmembrane region obtained by the TMpred server [64] (Fig 1C).

### Production and characterization of ANDV DIII and stem fragments

**Fig 2:**
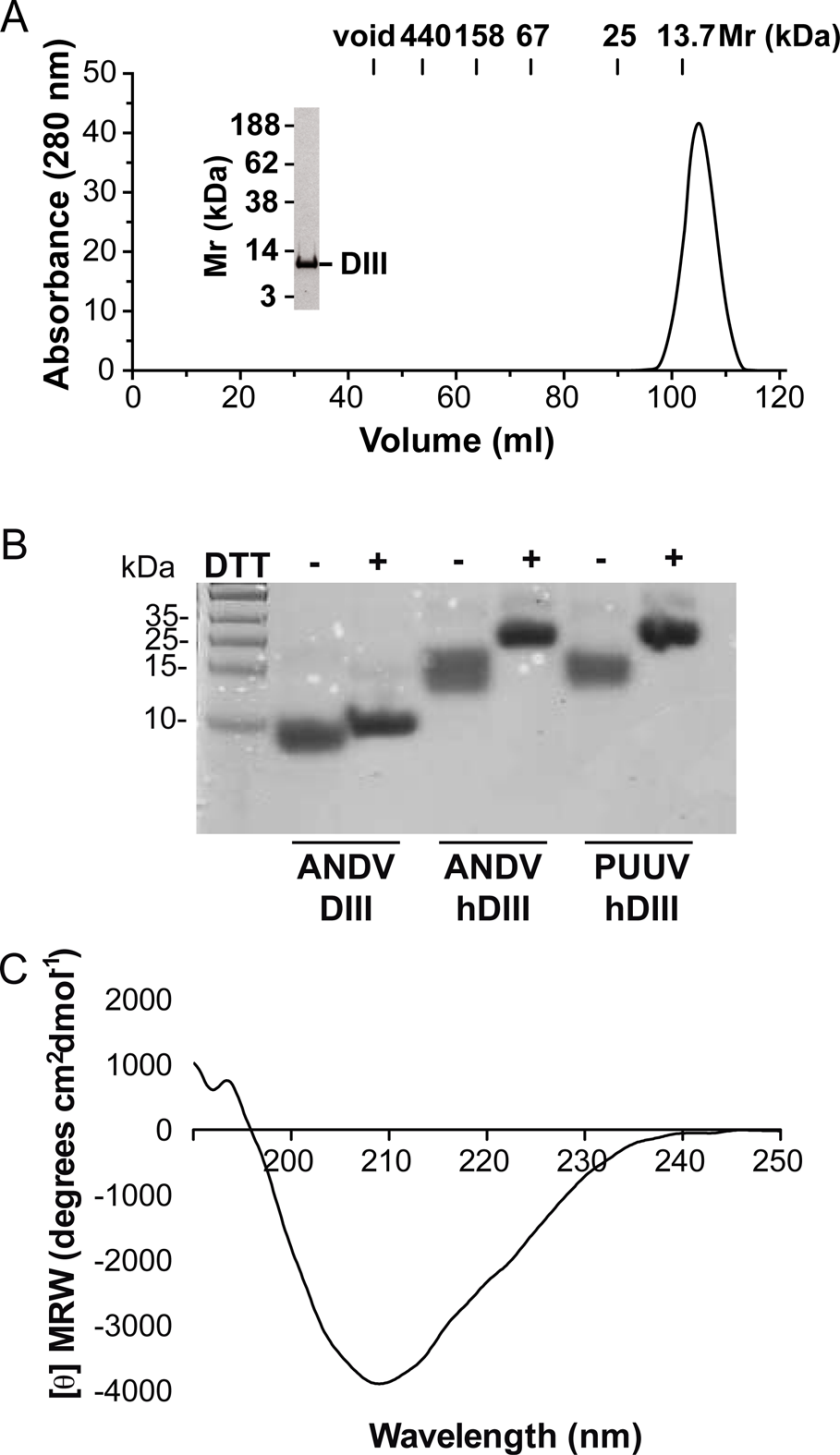
Characterization of recombinant DIII proteins. (A) Analysis of purified ANDV DIII by size-exclusion chromatography. The figure depicts the elution profile at 280 nm, and indicates the void volume and the elution volume of molecular size markers. The insert shows the analysis of an aliquot of the fraction corresponding to the chromatography peak, through SDS-PAGE followed by Coomassie staining. (B) Comparison of DIII proteins left untreated (-) or reduced (+) with 50 mM dithiothreitol followed by alkylation and western blot analysis. (C) Circular dichroism spectrum of ANDV DIII. The spectrum was acquired from 5 scans between 190 and 260 nm.

The production of DIII from different class II fusion proteins, including those of flaviviruses and alphaviruses, has been previously established in *E*. *coli* [52, 67–71]. The feasibility of preparing soluble DIII from flavi- and alphaviruses in a prokaryotic expression system retaining the native structure may be related to its globular IgG-like fold and lack of glycosylation [44]. The purification of DIII is generally achieved from inclusion bodies followed by refolding [44, 71], or from the supernatant of the cell lysate [52, 67–70]. Here, we prepared three recombinant DIII proteins; DIII derived from ANDV Gc with or without N-terminal His-tag (ANDV hDIII, ANDV DIII), and DIII from PUUV Gc with N-terminal His-tag (PUUV hDIII). The proteins were obtained from the supernatant of *E*. *coli* BL21 or Origami 2(DE3) cells, and eluted after their purification as a single peak detected by absorbance at 280 nm. Fig 2A shows an elution profile example of purified ANDV DIII from the last size exclusion chromatography column together with the homogeneity of the preparation assessed by SDS-PAGE. Because the predicted ANDV and PUUV DIII sequences contain eight highly conserved cysteine residues, the DIII proteins were next characterized for the presence of disulfide bridges by reduction and subsequent alkylation. An increase in the electrophoretic mobility could be detected for reduced ANDV DIII, ANDV hDIII and PUUV hDIII compared to its unreduced control (Fig 2B), indicating that the cysteines seemed to be arranged in disulfide bridges. We next explored the presence of secondary structure elements in DIII from hantaviruses by circular dichroism. The spectra showed a unique negative maximum at 209 nm (Fig 2C), confirming the presence of secondary structure. Deconvolution of the circular dichroism spectra into four components by different servers [54–56] indicated that DIII contained 40–41% β-sheets, ~60% random coils and turns with an α-helical content close to zero. This composition coincides with the high content of β-sheets and turns observed in DIII of class II fusion proteins [31]. Taking these data together, the monomeric form of recombinant ANDV DIII in solution, the presence of disulfide bridges, the secondary structure content, and the solubility of the recombinant protein (>20 mg/ml) indicate that DIII was folded.

In addition, we also produced a protein spanning the predicted DIII and stem region of ANDV Gc, including an N-terminal His-tag (ANDV hDIIIS). However, we did not obtain enough ANDV hDIIIS for its purification, presumably due to the poor solubility of this protein. To overcome this difficulty, we generated the predicted Gc stem region separately in two synthetic peptides, one spanning the N-terminal part (R1 peptide) and the other spanning the C-terminal part (R2 peptide) (Fig 1C). We also used a negative control peptide comprising a region of the ANDV nucleoprotein (NN peptide).

### Inhibition of ANDV cell entry by exogenous DIII and stem fragments

**Fig 3:**
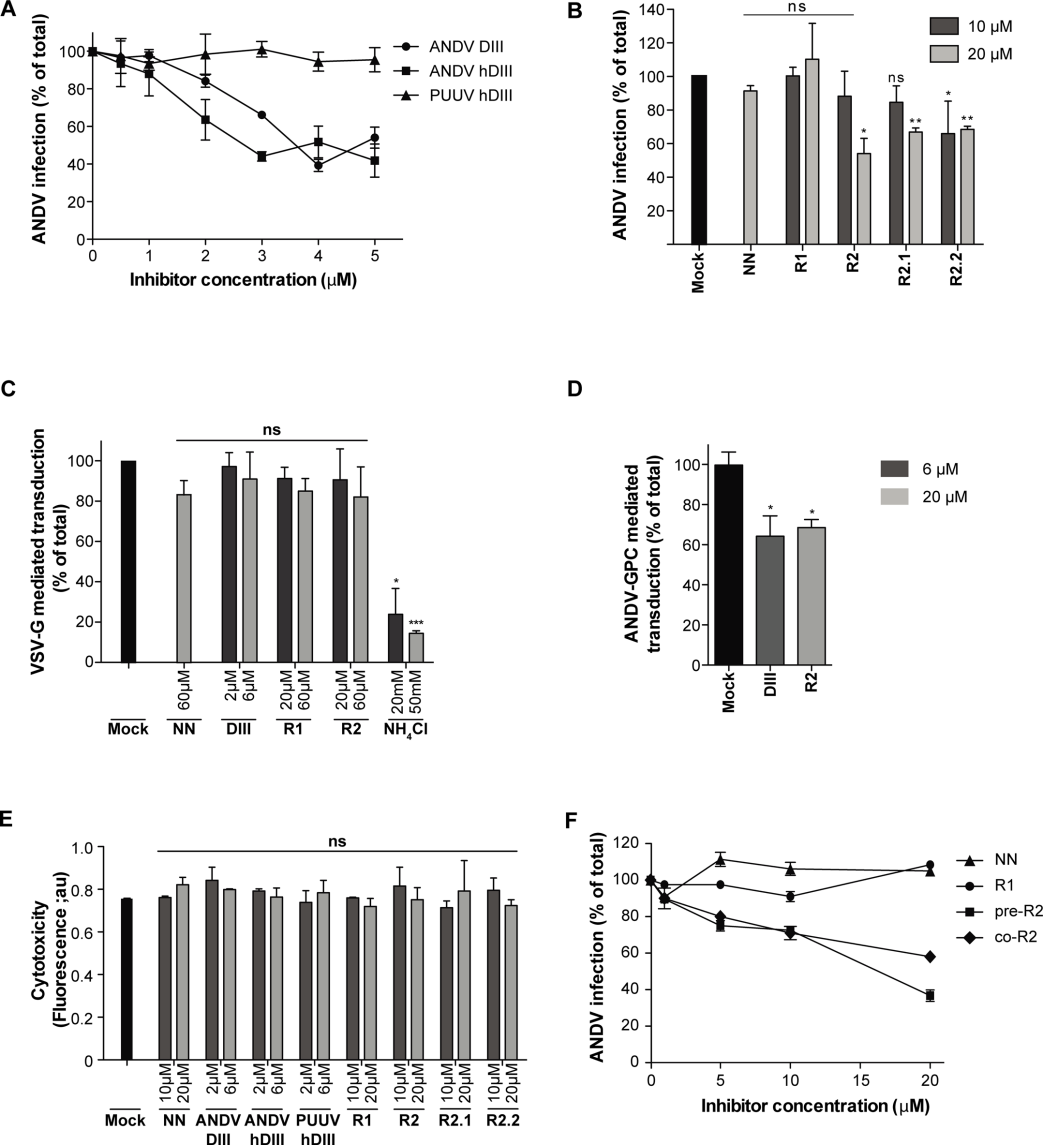
Exogenous DIII and stem fragments inhibit the infection of cells by ANDV. (A, B) Infection of Vero E6 cells by ANDV in the presence of exogenous DIII (A) or stem peptides (B). 100% infection was defined as the condition in the absence of Gc-derived fragments (Mock). (C, D) Transduction of Vero E6 cells by SIV vectors pseudotyped with VSV-G (C) or by SIV vectors pseudotyped with ANDV Gn/Gc glycoproteins (GPC; D) in the presence of exogenous DIII or stem peptides. Transduction was assessed by GFP reporter gene expression using flow cytometry, and 100% transduction defined as transduction in the absence of Gc-derived fragments (Mock). (E) Cytotoxicity analysis of Gc DIII proteins and stem peptides on Vero E6 cells. (F) ANDV inhibition by the R2 stem peptide through pre-incubation (pre-R2) or co-incubation (co-R2). For pre-incubation, ANDV was first incubated for 1 h with different concentrations of R2, and subsequently the mixture was incubated for 1 h with cells. For co-incubation, the virus and the R2 peptide were simultaneously added to the cells and incubated for 1 h. Infection was quantified in both cases by flow cytometry 16 h post-infection.

**Fig 4:**
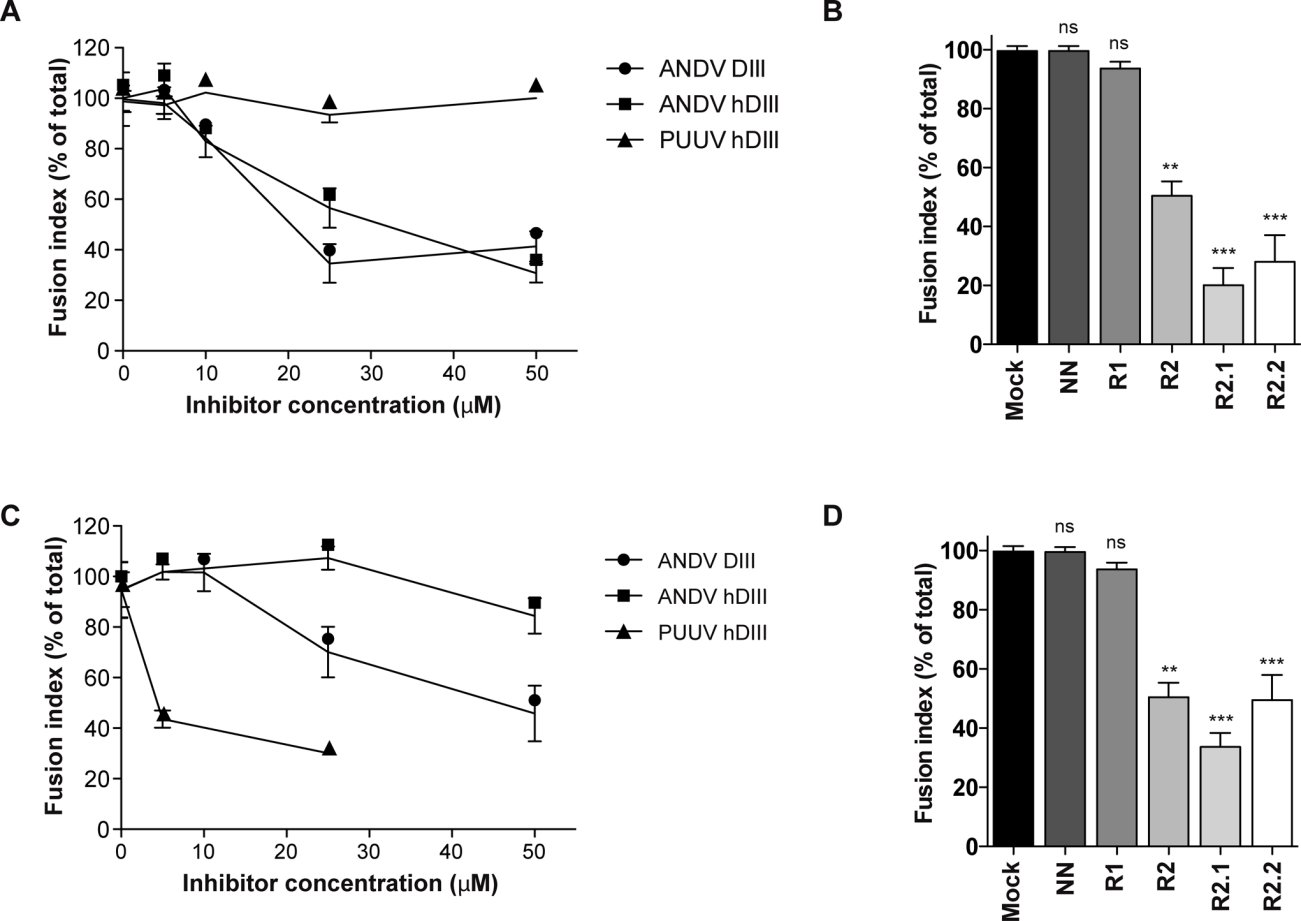
Exogenous DIII and stem fragments block glycoprotein-mediated cell-cell fusion. Inhibition of cell-cell fusion mediated by ANDV glycoproteins (A, B) and PUUV glycoproteins (C, D). The Gc fragments or controls were added during the 5 min of the low pH incubation step. The statistical evaluation of each data point was performed in relation to the Mock treatment: ***, P < 0.00025; **, P < 0.0025; *, P < 0.025; ns, not significant.

Once the predicted DIII and stem fragments were synthesized, purified, and characterized, we measured their inhibitory activity against ANDV during virus cell entry via the native, endosomal infection route. For this purpose, ANDV was incubated with Vero E6 cells in the presence of the Gc fragments. After 1 h incubation, the cells were washed and the infection monitored after 16 h based on an earlier established protocol [57]. ANDV DIII and ANDV hDIII reduced ANDV infection up to 60%, at 3–4 μM (Fig 3A). The N-terminal His-tag did not further improve inhibition of viral infection, as observed for the alpha- and flavivirus DIII proteins [44]. Interestingly, PUUV hDIII did not show any cross-inhibition of ANDV. This result was unexpected since cross-reactivity by DIII has been reported within the alphavirus and flavivirus genera [44, 72]. It is likely that the absence of cross-inhibition was due to the presence of the N-terminal His-tag in PUUV DIII (see results below; Fig 4C).

Next, we tested whether the ANDV stem peptides also impair ANDV infection. The R2 peptide, comprising the C-terminal part of the predicted Gc stem region, blocked the infection of cells by ANDV up to 55% at 20 μM (Fig 3B). In contrast, the R1 peptide spanning the N-terminal part of the predicted Gc stem, did not show any reduction of ANDV infection, similar to the negative control NN peptide (Fig 3B). To test whether a specific region of R2 was required for inhibition, we further tested two different parts of R2 (see Fig 1C). The N-terminal R2.1 and C-terminal R2.2 peptides both showed similar reducing effects on viral infection (Fig 3B).

To further explore the specificity of the Gc fragments on viral inhibition, we used as a model the unrelated vesicular stomatitis virus (VSV). While VSV enters cells by endocytosis and low pH-triggered fusion, this virus achieves fusion by a different class of fusion protein (class III). To analyze VSV-mediated entry, SIV vectors [24] were pseudotyped with the envelope glycoprotein G of VSV and the transduction of cells by this vector was evaluated by the expression of the GFP reporter gene. Neither ANDV DIII nor the ANDV stem peptides altered cell transduction at any tested concentrations up to 6 μM and 60 μM, respectively (Fig 3C). In contrast, when the pH of the endocytic route was neutralized with the weak base ammonium chloride, VSV-G mediated transduction was blocked up to 80% (Fig 3C). On the other hand, when SIV vectors were pseudotyped with ANDV glycoproteins, the DIII and R2 fragments produced ~40% and ~30% of inhibition of these vectors at 6 μM and 20 μM, respectively (Fig 3D), corroborating that the inhibitory fragments were active in this system.

None of the Gc-derived peptides and recombinant domains abrogated the viability of cells when they were incubated with Vero E6 cells (Fig 3E), indicating that the reduction of ANDV infection was not due to a cytopathic effect of the Gc-derived fragments.

It was previously reported that flavivirus stem peptides bind to the virus before it enters the cell, helping its delivery into endosomal compartments [47]. Based on this observation, we compared the inhibition of ANDV by (a) pre-incubation of the virus with the R2 peptide, or (b) co-incubating the R2 peptide with the virus during its adsorption to cells. Comparing the results of both experimental designs, a similar dose-dependent inhibition could be observed (Fig 3F), coinciding with the results obtained for flaviviruses (47). Based on this result, it seems plausible that binding of the R2 peptide to ANDV may occur very fast, making longer incubation times unnecessary.

Taken together, our data demonstrate that the exogenous fragments derived from ANDV Gc, comprising the predicted DIII or the C-terminal part of the predicted stem region, abrogate the entry of ANDV into cells.

### Exogenous DIII and stem fragments block cell-cell fusion

The inhibitory potential of the Gc fragments was next tested in a cell-cell fusion assay driven by the ANDV glycoproteins [22]. Therefore, we transfected Vero E6 cells with a plasmid coding for the ANDV glycoproteins, and incubated the cells 48 h post-transfection with low pH media for 5 min at 37°C, as described in Materials and Methods. The acidification of the media allows the activation of the viral Gc fusion protein on the plasma membrane of cells, thereby triggering the formation of syncytia. When the ANDV Gc fragments were incorporated in the low pH incubation step, ANDV DIII and ANDV hDIII diminished the glycoprotein-mediated fusion activity by ~70% at 25 μM and 50 μM, respectively (Fig 4A). In concordance with the results obtained with infectious ANDV (Fig 3A), the PUUV hDIII did not achieve cross-inhibition of ANDV glycoproteins (Fig 4A). On the other hand, the ANDV R2 stem peptides impaired the ANDV glycoprotein fusion activity up to 75% at 20 μM (Fig 4B).

### Cross-inhibition of hantaviruses by exogenous DIII and stem fragments

The ANDV-derived Gc fragments were likewise tested for their potential to cross-inhibit other hantaviruses such as PUUV. Therefore, we performed a cell-cell fusion assay driven by the PUUV glycoproteins. In this assay, PUUV hDIII blocked the PUUV glycoprotein-mediated fusion process, reducing fusion up to 65% at 25 μM (Fig 4C). This result coincides with the concentration range in which the ANDV DIII proteins block the ANDV glycoprotein fusion activity and confirms the activity of PUUV hDIII. When we tested the ANDV DIII proteins for cross-inhibition, we found that ANDV DIII, but not ANDV hDIII, blocked PUUV glycoprotein-mediated fusion up to 50% (Fig 4C). These results, together with the data on absent cross-reaction of PUUV hDIII with ANDV, suggest that the hantavirus DIII proteins without the N-terminal His-tag have cross-inhibiting activity; however the N-terminal His-tag of these domains seems to interfere in the inhibition. Thus so far, this notion remains to be corroborated with PUUV DIII lacking the N-terminal His-tag.

When we tested the ANDV stem peptides to block PUUV-mediated fusion, we found that they also had a cross-inhibition function (Fig 4D). Among them, the R2.1 peptide reached the highest cross-reduction result of ~70% at 20 μM, in line with the observation that this peptide also achieved the highest inhibition value of ANDV-mediated cell-cell fusion. Collectively, these results on hantavirus cross-inhibition suggest that residues in DIII and stem fragments are involved in intramolecular interactions. Some of these residues seem to be conserved between ANDV and PUUV (Fig 1D), allowing for the cross-interaction with exogenous DIII and stem fragments from a different hantavirus.

### Exogenous DIII and stem fragments block the fusion process

**Fig 5:**
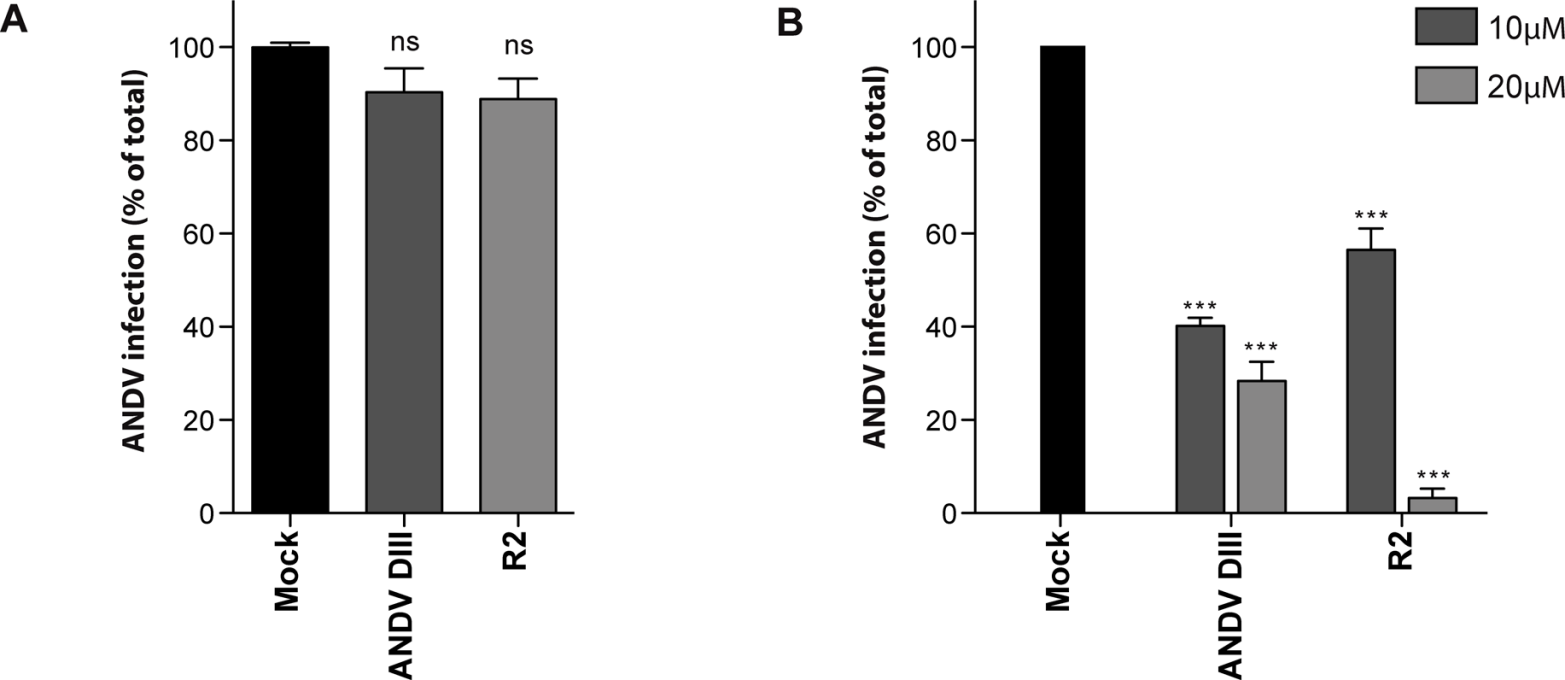
Exogenous DIII and stem fragments block the virus-cell membrane fusion process. (A) Incubation for 1 h of Vero E6 cells with exogenous DIII and stem fragments at 10 and 20 μM, respectively, followed by washing and subsequent inoculation with ANDV. (B) Inhibition of the fusion of ANDV with the plasma membrane by exogenous DIII and stem fragments. The Gc fragments or controls were added during the 5 min low pH incubation step. The statistical evaluation of each data point was performed in relation to the Mock treatment: ***, P < 0.00025; **, P < 0.0025; *, P < 0.025; ns, not significant.

ANDV cell entry can be blocked at different steps such as receptor binding and membrane fusion. For hantaviruses, the envelope glycoprotein or the specific domain involved in binding to receptors has not yet been identified. To discard a possible effect of the ANDV inhibitors in steps preceding virus-cell membrane fusion, we incubated the cells with ANDV DIII or the R2 peptide for 1 h before the addition of the virus. Unbound fragments were subsequently washed out, and cells were then infected with ANDV. The addition of neither DIII nor R2 at 10 and 20 μM, respectively, before the cells were incubated with ANDV led to a significant decrease in virus infection (Fig 5A). These data emphasize that the pre-incubation of DIII and stem fragments did not abrogate early steps in virus infection such as receptor binding or cellular signaling pathways. Furthermore, the results coincide with data obtained with stem peptides derived from DV2, where the pre-incubation of cells with these peptides also did not affect infection by DV2 [47].

Next, we asked whether the DIII and stem fragments interfered directly in the virus-cell fusion process. To test this, we assessed inhibition in a fusion infection assay, fusing ANDV with the plasma membrane. Therefore, ANDV was pre-bound to cells at 4°C and then ANDV DIII or R2 were added during the 5 min low pH pulse that triggers fusion. The blockade of this viral entry pathway was highly efficient in the case of the R2 peptide, reaching over 95% of inhibition at 20 μM (Fig 5B). Recombinant DIII led to a lower inhibition efficiency of 70% at 20 μM, result that in comparison to that obtained with R2 could be explained by the short incubation time in this experimental design. Compared to inhibition of the normal entry route of the virus, higher concentrations of ANDV DIII might have been necessary for inhibition since more input virus was used to reach similar levels of infection (MOI = 0.2). Taking these data together, our results confirm that the exogenous DIII and stem fragments function specifically during the viral fusion process.

### Exogenous DIII and stem fragments do not affect Gc homotrimer formation

**Fig 6:**
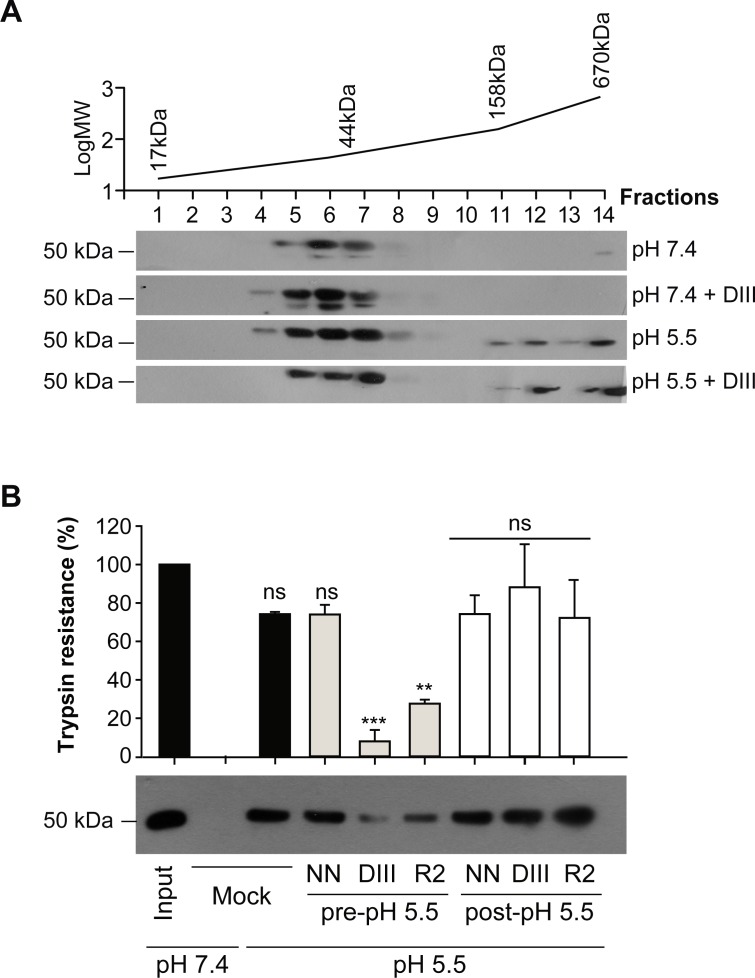
Exogenous DIII and stem fragments interfere with the formation of a stable post-fusion Gc trimer. (A) Low pH-induced multimerization changes of ANDV Gc in the presence or absence of exogenous DIII assessed by sucrose gradient sedimentation. ANDV was incubated for 30 min at pH 7.4 or pH 5.5, with or without ANDV DIII (6 μM). The presence of Gc in each fraction of the gradient was assessed by western blot analysis. The top panel indicates the sedimentation of molecular mass markers. (B) Gc homotrimer stability in the presence or absence of exogenous DIII and stem fragment R2. ANDV VLPs were incubated at the indicated pH and DIII (6 μM) or R2 (20 μM) added before or after acidification. After back-neutralization, the VLPs were treated with trypsin for 30 min and the digestion of Gc was assessed by western blot analysis. The trypsin resistance of Gc was quantified by densitometry (n = 3) and the statistical evaluation performed in relation to the input:***, P < 0.00025; **, P < 0.0025; *, P < 0.025; ns, not significant.

Since the fusion process involves multiple steps, we next assessed at which specific stage the Gc fragments interfere in this process. To this end, we started analyzing the trimerization of Gc using an earlier protocol [17]. ANDV was therefore incubated at neutral or low pH with or without DIII. Subsequently, the viral glycoproteins were extracted from the virus and applied to a sucrose gradient (7–15%) to evaluate their molecular mass. After ultracentrifugation, each fraction was examined by western blot analysis for the presence of Gc. Gradient sedimentation at pH 7.4 led to the detection of Gc in fractions corresponding to the molecular mass of Gc monomers of ~50 kDa (fractions 5–7), in the presence or absence of ANDV DIII (Fig 6A). Two Gc migration bands could be observed in the reducing electrophoretic system, which may correspond to different oxidation forms of Gc as described earlier [73]. Only the lower molecular mass band was previously found to shift from Gc monomers at neutral pH to Gc trimers at low pH [17]. When we incubated ANDV at pH 5.5, in the presence or absence of the DIII inhibitor, the lower molecular mass band of Gc shifted from the fractions corresponding to monomers and was found in fractions corresponding to Gc homotrimers (fractions 11–12; Fig 6A). This result indicates that ANDV DIII abrogated neither Gc fusion activation, nor Gc trimerization.

### Exogenous DIII and stem fragments prevent the formation of a stable Gc post-fusion trimer

The post-fusion conformation of ANDV Gc corresponds to a highly stable homotrimer [17]. In this context, we next explored the stability of the Gc homotrimer formed in the presence of exogenous DIII and stem fragments by assessing its resistance to protease digestion. For this experimental approach we used ANDV-like particles (VLPs) [62], since they can be purified to higher concentrations than the virus. These VLPs resemble the viral envelope by exposing the Gn and Gc glycoproteins. To test their trypsin resistance, the VLPs were first incubated at neutral or low pH in the presence or absence of the inhibitors, and subsequently subjected to digestion with trypsin for different incubation times. As expected, the neutral pH form of Gc was fully degraded within 30 min, while the low pH form of Gc was largely resistant to digestion (Fig 6B). In contrast, Gc lost its trypsin resistance and digestion could be observed when ANDV DIII or the R2 peptide was co-incubated with VLPs during the low pH incubation step, indicating that they interfered in the formation of a stable Gc homotrimer (Fig 6B). Some residual Gc could be detected, most likely because the DIII or stem fragments did not block all Gc molecules, which coincides with the inhibitory results (Figs 3A and 3B and 5B). The formation of a stable homotrimer was however not prevented when the control peptide NN was added before acidification, nor when either the NN peptide, DIII or the R2 peptide was added after the low pH incubation, confirming the specificity of the assay. Together, these data suggest that the exogenous DIII and stem fragments prevented the formation of a stable post-fusion hairpin structure, presumably by direct binding to Gc in its extended intermediate conformation.

### Exogenous DIII arrests the fusion process in a step preceding the hemifusion intermediate

**Fig 7:**
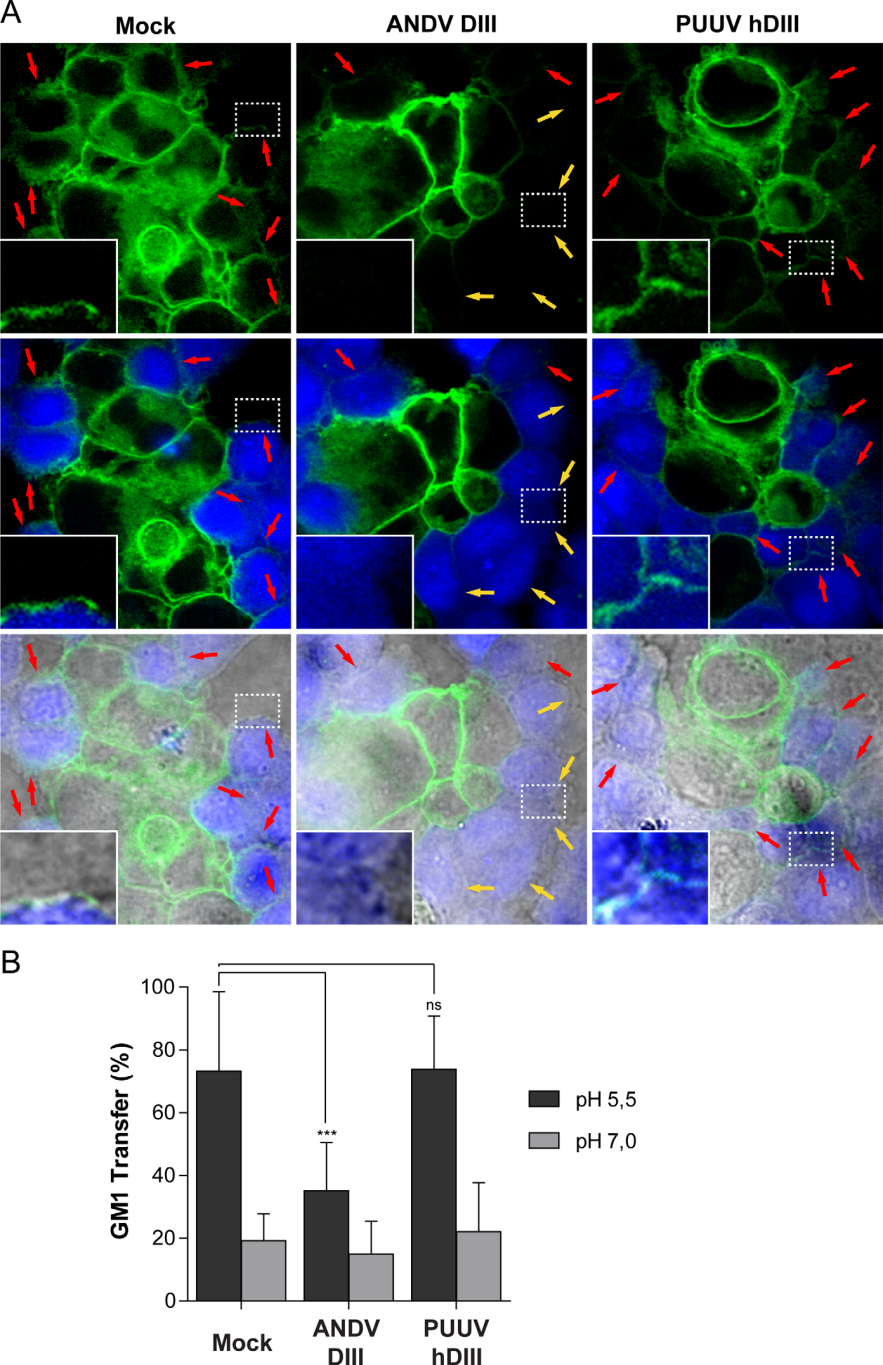
Exogenous DIII and stem fragments block a fusion stage preceding the mixing of outer membrane leaflets. (A) Fluorescence confocal microscopy of the GM1 transfer from ANDV glycoprotein-expressing 293FT effector cells (GM1^+^) to CHO-K1 target cells (GM1^-^), treated at pH 5.5. CHO-K1 cells were stained with CMAC (blue fluorescence) and GM1^+^ cells were labeled with the cholera toxin β-subunit conjugated to Alexa Fluor 488 (green fluorescence). Red arrows indicate GM1 transfer to CHO-K1 target cells (resulting in GM1^+^); yellow arrows indicate CHO-K1 target cells negative for GM1 (remaining GM1^-^). Magnification 60x. (B) Quantification of the GM1 transfer between ANDV glycoprotein-expressing 293FT cells (GM1^+^, effector cells) to CHO-K1 cells (GM1^-^, target cells), in the absence (Mock) or presence of recombinant ANDV DIII or PUUV hDIII. CHO-K1 cells were only considered GM1^+^ when the GM1 signal was detected at the full circumference of the cells. ***, P < 0.00025; **, P < 0.0025; *, P < 0.025; ns, not significant.

The presence of exogenous Gc fragments interfered neither with the activation of ANDV Gc nor with its homotrimerization; however it did not allow the formation of a stable post-fusion structure. The hemifusion intermediate is a stage that occurs between these fusion steps, in which the outer membranes of the virus and cell have fused, while the inner leaflets still remain apart. In order to test if the inhibition of ANDV by exogenous DIII occurs before or after the hemifusion intermediate state, we developed a hemifusion assay for the ANDV glycoproteins. For this purpose, we took advantage of a previously developed cell-cell fusion assay between 293FT cells expressing the ANDV glycoproteins (effector cells) and CHO-K1 cells (target cells) [17]. In this assay, the low pH-triggered transfer of GM1 from effector cells to target cells was analyzed by confocal microscopy, which allows detection of GM1 at the cell surface in a single focal plane, with minimal sample intervention (Fig 7A). To allow unambiguous identification of GM1 transfer from effector cells to target cells, CHO-K1 cells were only defined as GM1^+^ when the label was detected on their full circumference (Fig 7A, red arrows). Conversely, target cells in contact with effector cells, but showing only partial or no GM1 stain, were defined as GM1^-^ (Fig 7A, yellow arrows). Although this assay allows for the detection of lipid mixing between cells, it does not discriminate between full fusion and hemifusion of membranes. To obtain a quantitative measure of GM1 transfer from effector to target cells, we quantified the percentage of transfer in each condition. At pH 7, a background level of transfer around 20% was observed in all conditions (Fig 7B). When glycoproteins were activated at pH 5.5, GM1-transfer from effector cells to target cells was detected in ~70% of Mock control treatment (Fig 7B). However, when ANDV DIII was incorporated in the low pH incubation, the transfer of GM1 appeared to be less frequent, decreasing to below 40% (Fig 7B). This value is still higher than the background level observed for GM1 transfer at neutral pH, indicating that this domain reduced lipid mixing in an incomplete manner. Most probably, exogenous ANDV DIII did not make contact with all fusion proteins during the short low pH incubation, which coincides with the results for blocking cell-cell fusion (see Fig 4A). The impairment of GM1-transfer was highly specific, since the incorporation of PUUV hDIII, which has no cross-inhibition activity against ANDV (see Figs 3 and 4A), did not abrogate the acid-induced GM1 transfer of ~70% (Fig 7A and 7B). In summary, our results show that ANDV DIII arrests the fusion process after Gc trimerization, but before reaching a hemifusion intermediate. In this context, it seems likely that Gc fragments prevent the movement of the endogenous DIII or the stem region towards the core of the Gc trimer as described for other class II fusion proteins [39, 44].

## Discussion

The fusion of the viral membrane with a host cell membrane is a crucial step in the entry of enveloped viruses into cells. In the present study we demonstrated that predicted DIII and stem fragments blocked acid-induced fusion of ANDV within the endosomal entry pathway and with the cell surface. Fusion was allowed to proceed until Gc trimerization, but prevented membrane hemifusion and fusion pore formation. These results not only provide novel information about inhibitory strategies against ANDV and other hantaviruses, but also provide a proof of concept that Gc shares structural similarity with the overall fold of class II fusion proteins.

Comparing the inhibition of hantaviruses by exogenous DIII with that of other class II fusion proteins, ANDV and PUUV hantaviruses were blocked by ANDV DIII without additional N-terminal His-tag or C-terminal residues, although containing seven N-terminal residues derived from the GST-tag. While the addition of an N-terminal His-tag achieved a 100-fold improvement in blocking fusion of Semliki Forest virus [44], ANDV DIII ‒ with or without N-terminal His-tag ‒ achieved similar inhibitory results. For SFV DIII it has been proposed that this N-terminal tag may mimic the domain I-DIII linker region, thereby stabilizing the interaction with the fusion protein core [44]. On the other hand, it has been reported that the presence of C-terminal residues derived from the stem region is necessary for inhibition by exogenous DIII of DV2 and chikungunya virus E proteins [44, 72]. More specifically, for chikungunya virus it has been shown that nine residues from the E stem region are required for DIII to bind to the fusion protein domain I-domain II core. Hence, the potency of inhibiting the ANDV fusion process through ANDV and PUUV DIII may be further improved in future studies by adding N- or C-terminal residues.

The ANDV DIII, including the stem region, was largely insoluble and therefore this larger Gc fragment could not be tested in the ANDV entry assays. The R1 peptide spanning the first 17 N-terminal residues of the 44-residue stem region did not affect ANDV entry, while the R2 peptide spanning the last 20 C-terminal residues blocked ANDV infection to a similar extent as DIII. When we tested the two peptides R2.1 and R2.2, comprising either the N- or C-terminal half of the ANDV R2 stem peptide, the inhibitory activity against ANDV was largely retained by both peptides. Hence, the R2 peptide seems to contain residues in these two regions that participate similarly during inhibition. Similar to the C-terminal region of the ANDV stem region, the C-terminal region, but not the N-terminal, of DV2 E protein binds and blocks the fusion protein [47]. Interestingly, inhibitory peptides derived from membrane proximal regions (or stem regions) of diverse fusion proteins such as those of RVFV, DV2, SARS-coronavirus, Influenza virus, and Hepatitis C virus have been found likely to interact with membrane interfaces by a hydropathy segment [74], which is predicted by the Wimley-White interfacial hydrophobicity scale (WWIHS) for the transfer of a peptide from an aqueous environment to a palmitoyl-oleoyl-phosphatidyl choline interface [75, 76]. A need for such a membrane-binding property for inhibitory activity coincides with studies on peptides derived from the stem region of alphavirus fusion proteins; these peptides generate WWIHS below values of 1 (S1 Table), indicative of weak membrane binding [74], and fail to block alphavirus fusion activity [44, 72]. In the case of the DV2 E protein, such a membrane-binding sequence is found at the C-terminal end of the peptide [48] with a high WWIHS value (S1 Table). In fact, the stem peptides of the DV2 E protein have been shown to interfere with the fusion of the virus in the endosomal compartment by a two-step mechanism: first by binding to the viral membrane outside the cell, and next by binding against the E core trimer once fusion has been triggered in the endocytic compartment (47). Using the Wimley-White scale [77], an interfacial hydropathy segment with a WWIHS vale >5 was predicted for the ANDV Gc stem region that coincides with the sequence of the R2 peptide (S1 Table), indicative for moderate membrane partitioning [74]. If the ANDV R2 peptide would interact with membranes by a hydropathy segment, then such an interaction did not favor ANDV inhibition sufficiently in the endosomal route, since fusion impairment was most efficient when directly present in the fusion compartment; when applied at the same concentration, the R2 stem peptide blocked 45% of ANDV infection when entry occurred via the endocytic pathway, while R2 reached over 95% inhibition of ANDV infection when fusion occurred at the plasma membrane. In this sense, modifications to the R2 peptide that favor membrane interaction, such as those introduced to peptides derived from the West Nile virus E stem region [48] or the membrane proximal region of Influenza virus hemagglutinin and HIV gp41 [78, 79], may help in the future to improve its inhibitory activity and to direct it towards the closed environment of endosomes.

The ANDV DIII and stem peptides blocked not only fusion mediated by ANDV glycoproteins, but also the fusion activity of the glycoproteins of another hantavirus (PUUV). This result is in accordance with the high sequence identity of 72% between DIII of these viruses and also with reports on the cross-inhibition activity of DIII within the genus *Alphavirus* [72], where DIII conservation is as high as 50%. However, the N-terminal His-tag of ANDV hDIII seemed to prevent the cross-inhibition of PUUV fusion activity, indicating that this tag may interfere in specific binding to Gc from heterologous species. It is likely that the histidines of the tag become positively charged in the low pH environment, which in turn may induce repulsion with positively charged residues in the Gc of hantaviruses. Such repulsion may be overcome by a higher binding affinity of DIII to Gc from the same hantavirus, but not to heterologous viruses. In addition to the DIII of ANDV, peptides derived from the stem region of ANDV also cross-inhibited the PUUV fusion activity, which further corroborates the presence of conserved residues among hantavirus Gc proteins that are involved in the likely binding of this peptide. Cross-inhibition of fusion proteins by stem peptides has been previously reported for viruses from the genus *Flavivirus*; Dengue virus stem peptides blocked different Dengue virus serotypes but not other flaviviruses. [48]. The absence of cross-inhibition in that case was related not to a poor interaction with the respective E protein, but rather to a poor interaction with the viral membrane [48]. Finally, stem peptides derived from the RVFV Gc protein have been reported to block the three different classes of viral fusion proteins [46], acting as a broad-spectrum fusion inhibitor [80]. For the exogenous stem peptides from ANDV we did not observe cross-inhibition of other fusion proteins such as that of VSV at concentrations up to 60 μM. Therefore, it is more likely that the ANDV stem fragments may be applied to inhibit similar viruses within the same genus, but not other viral fusion machineries.

Taken as a whole, our results demonstrate that strategies employed against class II fusion proteins allow for the inhibition of hantaviruses such as ANDV and PUUV. Although targeting the endosomal site of virus fusion has not yet been optimized, it was possible to block fusion and infection under physiological virus entry conditions. Hopefully, the novel inhibitory strategy based on ANDV DIII and stem peptides will help in the future development of therapeutic strategies against different hantaviruses.

## Supporting Information

S1 Table Prediction of hydropathy segments. The hydropathy segments were predicted for the stem region of alpha-, bunya-, and flavivirus fusion proteins using the Wimley-White interfacial hydrophobicity scale (WWIHS). Rift Valley Fever virus (RVFV), Dengue virus type 2 (DV2), Semliki Forest virus (SFV), Chikungunya virus (CHIKV).
(DOCX)
