# Supplementary material for: Inhibition of the Hantavirus Fusion Process by Predicted Domain III and Stem Peptides from Glycoprotein Gc
Source: PLoS Negl Trop Dis. 2016 Jul 14;10(7):e0004799. doi: 10.1371/journal.pntd.0004799 (PMC4945073; doi:10.1371/journal.pntd.0004799)
Supplement: S1 Table — The hydropathy segments were predicted for the stem region of alpha-, bunya-, and flavivirus fusion proteins using the Wimley-White interfacial hydrophobicity scale (WWIHS). Rift Valley Fever virus (RVFV), Dengue virus type 2 (DV2), Semliki Forest virus (SFV), Chikungunya virus (CHIKV). (DOCX) [file pntd.0004799.s001.docx]

**S1 Table.** **Prediction of hydropathy segments.** The hydropathy segments were predicted for the stem region of alpha-, bunya-, and flavirus fusion proteins using the Wimley-White interfacial hydrophobicity scale (WWIHS). Rift Valley Fever virus (RVFV), Dengue virus type 2 (DV2), Semliki Forest virus (SFV), Chikungunya virus (CHIKV).

| **Viral fusion protein** | **GenBank accession no.** | **Hydropathy-predicted segment** | **WWIHS score*** |
| --- | --- | --- | --- |
| ANDV Gc | AAO86638 | CTFKCWFTKSGEWLLGILN | 5.61 |
| RVFV Gc | P21401 | SWNFFDWFSGLMSWFGGPL | 10.20 |
| DV2 E | AIU47320 | ILGDTAWDFGSLGGVFTSI | 5.17 |
| SFV E1 | P03315 | ASHSNVVFPDMSGTALSWV | 0.75 |
| CHIKV E1 | 2V33_A | ASHTTLGVQDISATAMSWV | 0.01 |

* Calculated at the fusion pH, were histidine is charged and aspartate and glutamate uncharged.
